# SARS-CoV-2 Survival on Surfaces. Measurements Optimisation for an Enthalpy-Based Assessment of the Risk

**DOI:** 10.3390/ijerph20126169

**Published:** 2023-06-18

**Authors:** Angelo Spena, Leonardo Palombi, Mariachiara Carestia, Vincenzo Andrea Spena, Francesco Biso

**Affiliations:** 1 Department of Enterprise Engineering, Tor Vergata University of Rome, 00133 Rome, Italy; spena@uniroma2.it; 2 Catholic University of “Our Lady of Good Counsel”, 1001 Tirana, Albania; palombi@uniroma2.it; 3 Department of Biomedicine and Prevention, Tor Vergata University of Rome, 00133 Rome, Italy; mariachiara.carestia@uniroma2.it; 4 Department of Astronautical, Electrical and Energy Engineering, Sapienza University of Rome, 00184 Rome, Italy; vincenzo.spena@uniroma1.it

**Keywords:** viruses survival, SARS-CoV-2, virus stability on surfaces, measurements improvement, specific enthalpy

## Abstract

The present work, based on the results found in the literature, yields a consistent model of SARS-CoV-2 survival on surfaces as environmental conditions, such as temperature and relative humidity, change simultaneously. The Enthalpy method, which has recently been successfully proposed to investigate the viability of airborne viruses using a holistic approach, is found to allow us to take a reasoned reading of the data available on surfaces in the literature. This leads us to identify the domain of conditions of lowest SARS-CoV-2 viability, in a specific enthalpy range between 50 and 60 kJ/Kg_dry-air_. This range appears well-superimposed with the results we previously obtained from analyses of coronaviruses’ behaviour in aerosols, and may be helpful in dealing with the spread of infections. To steer future investigations, shortcomings and weaknesses emerging from the assessment of viral measurement usually carried out on surfaces are also discussed in detail. Once demonstrated that current laboratory procedures suffer from both high variability and poor standardisation, targeted implementations of standards and improvement of protocols for future investigations are then proposed.

## 1. Introduction

Studies on SARS-CoV-2 during the pandemic confirmed that possible transmission routes are direct contact, aerosols, and fomites. Using a holistic approach to investigate how temperature and humidity simultaneously affect the vitality of airborne viruses, since the beginning of the pandemic we proposed a method [1] based on the thermodynamic property Enthalpy, formerly introduced by the Dutch physicist H. Kamerlingh Onnes as H = U + pV, i.e., the sum of the internal energy U of a system and the product of its pressure p and volume V. As a matter of fact, during a process occurring at constant pressure, the Enthalpy variation represents the overall heat (sensible + latent) exchanged by the system, thus allowing us to define its state with just one parameter, which brings together information about both temperature and water content (humidity). The method can be used to analyse the results of literature or research experiments aimed at investigating the relationship between pathogens and environmental conditions; and, more importantly, to better design the ambient air parameters to assess the survival patterns.

Indeed, when dealing firstly with the coronaviruses viability in aerosols with the aim of understanding how to mitigate the virulence of SARS-CoV-2 by maintaining adverse conditions in indoor environments, specific enthalpy *h* (i.e., Enthalpy per unit mass) was found [1] to be correlated with virus survival. The method was then also successfully used to attempt a relationship between SARS-CoV-2 infectivity and outdoor climatic conditions, leading to an enthalpy-related seasonal risk scale [2] to predict the potential danger of the spread. It is worth noting that, until then, the scientific literature investigating the survival of viruses in air had not been able to provide unambiguous indications when temperature or humidity varied separately [3], sometimes finding trends that contradicted the experimental evidence collected over time.

Assessing whether the domain identified of higher survival and infectivity in aerosols, which falls in an enthalpy range of 50 to 60 kJ/Kg_dry-air_, is superimposable with that on surfaces, could allow for a general model of the survival of viruses in the environment. This result could steer future investigations and provide valuable indications for facing the spread of infections. The method can also guide the correct design of setup/setting of HVAC facilities to reduce the risk of indoor infection. Moreover, we can also use the method as an index to predict the risk linked to outdoor climatic variations thus supporting decision-makers in selecting the most appropriate social actions.

However, a number of physical and environmental parameters influencing the survival of viruses within their envelope could interfere with sampling during virus viability measurements in the laboratory environment. In addition to temperature and relative humidity, the pH value, the presence of pollutants, and UV [4] radiation can be decisive. Therefore, testing on surfaces from which the virus must be removed with its envelope for measurement purposes entails reducing the disturbance of confounding factors arising during the handling of samples.

In this frame, the present work has made it possible not only to prove the general validity of the enthalpy range previously established for aerosols but also, at the same time, to identify relevant criticalities of experiments on surfaces and to suggest improvements in their measurement procedures.

## 2. Materials and Methods

For the present work, an extensive search of peer-reviewed publications, which dealt with the survival of SARS-CoV-2 and reported on its half-life by modelling the phenomenon with an exponential decay equation, was performed. To do this, the works based on the two-phase decay model, which results from the sum of a fast and a slow exponential decay [5,6], were excluded. As a matter of fact, directly correlating the published half-lives relating to the two stages of decay to the half-life relating to a single-phase model would theoretically be possible, but reprocessing the original data could lead to additional noise to the results produced by the authors. Moreover, not all types of data needed for our purpose were always available in publications. It was therefore preferred, comforted by the small number of works based on the two-phase model, to exclude the latter from the analysis. The articles consulted that did not report half-life as a summary parameter were also not included in the present discussion [7,8,9,10,11,12].

**Figure 1:**
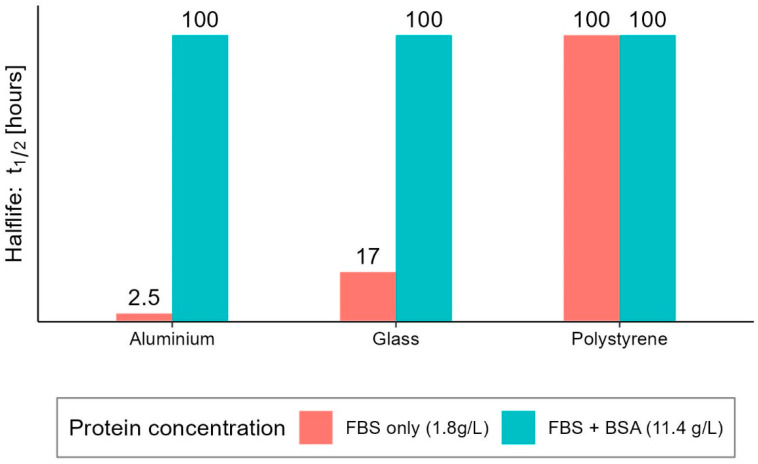
Effect of protein concentration of culture medium on SARS-CoV-2 survival on aluminium, glass and polystyrene surfaces under the same environmental conditions. Visualisation generated from the data published by Pastorino et al. [29]. The authors reported half-lives greater than 96 h as maximum values, here conventionally reported as 100 h for visualisation purposes. FBS: foetal bovine serum; BSA: bovine serum albumin; final protein concentration in parentheses.

**Figure 2:**
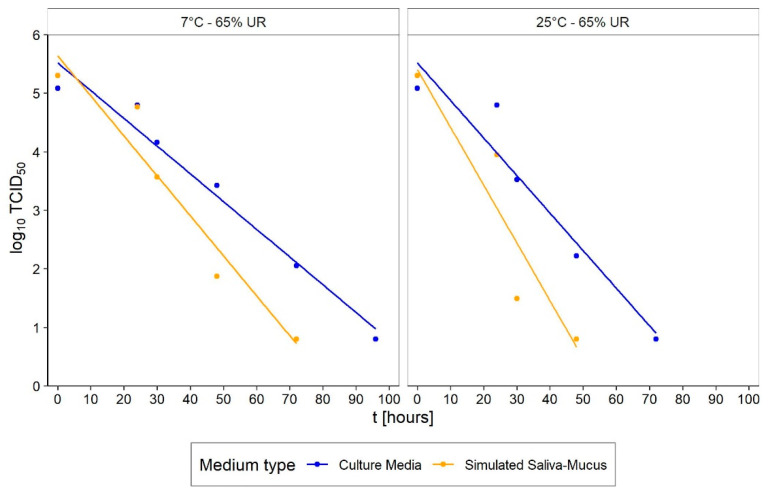
Effect of medium on SARS-CoV-2 survival on stainless steel surface under different environmental conditions. Elaboration generated from the data published by Szpiro et al. [30].

**Figure 3:**
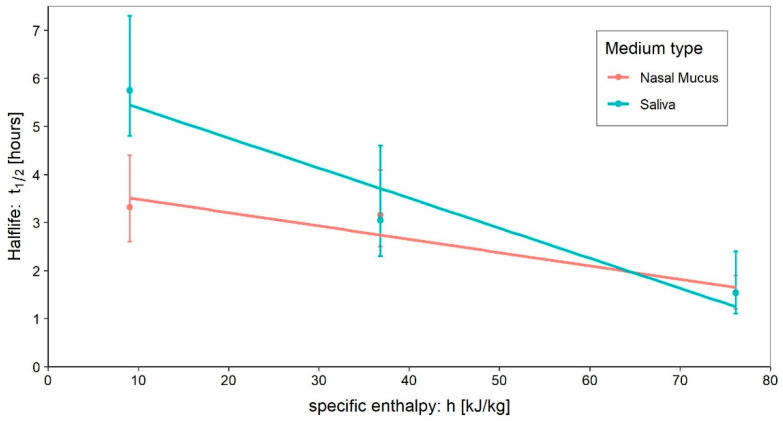
Effect of medium on SARS-CoV-2 survival on polystyrene surfaces under different environmental conditions. The analysis was carried out using the data published by Matson et al. [31]. The data are plotted with a small amount of jitter due to the presence of the same values of half-lives at intermediate enthalpy values (around 35–40 kJ/kg_dry-air_).

The survival studies we analysed can be grouped as follows: (i) those that investigated the dependence of virus survival on the type of surface; and (ii) those that investigated it as the temperature or relative humidity varied. The former set generally provides survival data for a fixed setup of environmental parameters as the surface type changes, while the latter set provides information on the interaction between the virus and the environment. More specifically, considering that differences in the aminoacidic composition and sequence (and therefore in the tridimensional structure) influence the behaviour of the protein in response to changes in the surrounding physical and chemical environment, it is worth noting that:

- *Relative humidity, RH:* is believed to be responsible for greater or lesser stability of viruses with a lipidic envelope [13,14], albeit the presence of specific proteins jointly influences the envelope stability [15,16]
- *Temperature*: is generally most investigated by holding relative humidity constant and is believed to be responsible for stabilising the lipidic layer at low temperatures and high humidity [17]. Furthermore, low temperatures and low relative humidity favour the survival and transmission of certain influenza viruses [18,19], and are associated with an increased occurrence of respiratory tract infections.
- *pH*: is believed to be responsible for changes in the survival of enveloped viruses, as it causes alterations in viral glycoproteins that result in a limited ability to infect [20,21]. Furthermore, while viability decreases in saline solutions, it increases significantly in mucous [22]
- *UV light*: action on viruses is well known in the literature, and sterilisation by UV light is a commonly used process. Although several studies have already demonstrated the effect of UV on SARS-CoV-2 [23,24,25,26], controlling the exposure of samples to light during experiments is not always carried out, which becomes an additional confounding factor and a source of data dispersion. This circumstance affects the validity of comparing the results of different experiments.
- *Medium*: different media, or the variation in their composition, is another confounding factor [26,27,28]. The protein composition of the medium, for example, alters the ability of the virus to proliferate and survive, as demonstrated by Pastorino et al. [29] (Figure 1). This constitutes a further obstacle when comparing data from different experiments. We can perceive this dependence appropriately by visualising the data from the work of Szpiro et al. [30] (Figure 2) and Matson et al. [31] (Figure 3), the latter expressly given as a function of specific enthalpy.
- *Pollutants*: the opinion of the scientific literature is now converging on the established role of pollutants in the survival and transmission capacity of viruses [32,33]. However, this role is especially significant when studying the survival of viruses in aerosols; it does not appear to be relevant—as was also the case in the present study—for the survival of SARS-CoV-2 on surfaces.

A critical point that emerges when analysing the work on the survival and viability of SARS-CoV-2 on surfaces, is the great variability among the parameters of the laboratory setup. As also highlighted by Bueckert et al. [28], we can see differences in: (i) the type and composition of the culture medium; (ii) its volume; (iii) the strain of the virus under investigation; (iv) the substrate and the method of titre quantification (PFU, TCID50). All these varying factors contribute to generating the noise observed when comparing data from different authors. In this regard, a comparison with the procedure used to measure the survival of the virus in aerosol showed that the latter has greater standardisation and generates less noise, allowing for a better results comparison. Indeed, the variability of decay data, especially in surface tests, is a criticality that has already been reported in the literature [34].

The present work uses the Enthalpy method to identify infectious risk domains. As in our previous studies [1,2], for each thermodynamic equilibrium state identified by its temperature and relative humidity, the specific enthalpy of moist air *h* has been calculated as follows [35]:

h = c_a_t + AH(c_v_t + r), (1)

AH = 0.623 [(RH%/100) p_s_(t)]/[p − (RH%/100) p_s_(t)] (2)

where c_a_ and c_v_ are, respectively, the specific heat at a constant pressure of dry air, and of water vapour, which, around ambient temperature, can be assumed to be correspondingly equal to 1.006 kJ/kg°C and 1.86 kJ/kg°C; t is the temperature in centigrade degrees; AH is the absolute humidity of moist air, in kg_v_/kg_dry-air_, also called *humidity ratio* and defined as the ratio of the mass of water vapour to the mass of dry air in the moist air sample; r is the latent heat of vaporisation of water at its triple point, equal to 2501 kJ/kg; p_s_(t) is the saturated vapour pressure of water at temperature t in Pascal; and p is the total pressure of moist air, typically the atmospheric pressure, in Pascal.

**Figure 4:**
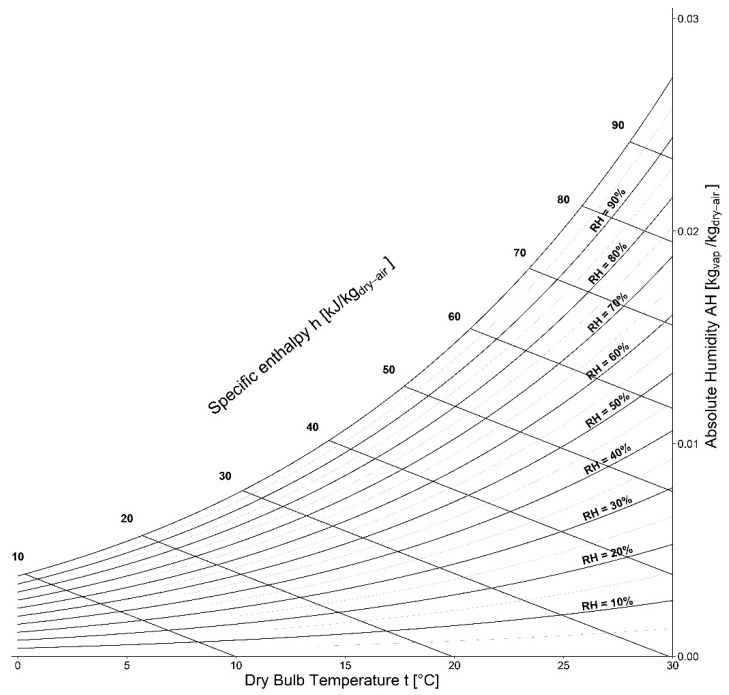
Psychrometric chart in a relevant temperature domain.

The saturated vapour pressure of water in Pascal can be calculated from the empirical formula derived by Hyland and Wexler for the temperature range of 0 to 200 °C [35,36]:

ln(p_s_(T)) = C_1_/T + C_2_ + C_3_T + C_4_T^2^ + C_5_T^3^ + C_6_ln(T) (3)

in which C1 = −5.8002206 × 10^3^, C2 = 1.3914493 × 10^0^, C3 = −4.8640239 × 10^−2^, C4 = 4.1764768 × 10^−5^, C5 = −14452093 × 10^−8^, C6 = 6.5459673 × 10^0^, whereas T is the absolute temperature in Kelvin degrees, namely T = t + 273.15. In Figure 4, a map (in terms of psychrometric chart) of the most significant environmental conditions occurring at the ground in terms of indoor or outdoor thermodynamic states of equilibrium is reported.

In the present work, we performed both linear and polynomial linear regressions; the level of significance was established at *p* < 0.05; the analysis was carried out in R version 4.2.1; figures were produced using the package ggplot2.

## 3. Results

The synthetic parameter here assumed to summarise the response of the virus to experimental conditions is its half-life. For it, reference was made to the values published by the various authors in the reviewed articles. Data on virus survival over time in the examined works were evaluated in terms of Plaque Forming Units (PFU) or the Median Tissue Culture Infectious Dose (TCID50). The two parameters can be related to each other as long as assumptions about cell lines and titration protocols are verified [37]. The use of the half-life parameter allowed us to overcome the problem, making the phenomenon directly comparable without the risk of introducing an additional source of uncertainty.

Table 1 summarises all the data collected from the reviewed literature and the calculated specific enthalpy values.

**Figure 5:**
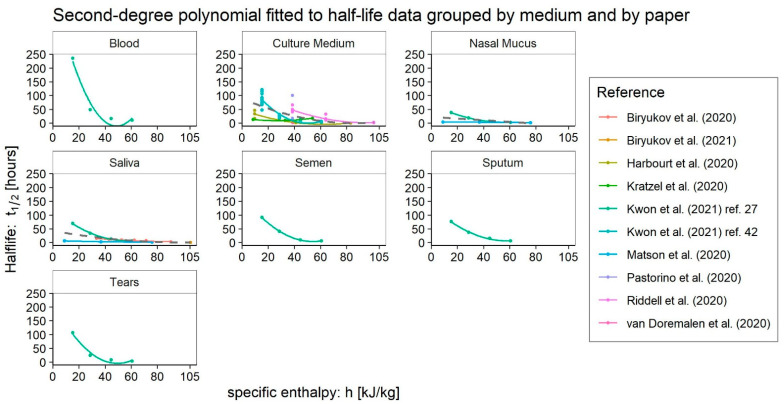
Comparison of SARS-CoV-2 survival data from different available works [27,29,31,34,38,39,40,41,42,43], grouped by medium. The grey dashed lines are second-degree polynomial regressions of all available data points for each medium.

The available data were analysed, given the related dependence of virus survival measures, according to the medium chosen for experimentation. In addition: (i) from the papers that explored fewer than three different environmental conditions, only the points are reported; (ii) from the papers that explored three (minimum number of points required) or more environmental conditions, fitting was restricted here to a second-degree polynomial (Figure 5); (iii) eventually, a third-order curve will be attempted later (Figure 7) once all the points from the different authors have been aggregated by medium type.

As with other coronaviruses, experimental evidence confirmed the improved surface survival of the virus at low temperatures and, consequently, specific enthalpies [3,27,28,30,31,42,43,44]. Indeed, when analysing the data displayed in Figure 5, we can see that almost all the works are in good accordance with the expected behaviour of the virus. However, some exceptions arise. First, Cappi et al. [45] dispute the possibility of defining a recurrent seasonal pattern for SARS-CoV-2. Yet, the finding about a missing seasonal pattern could be related to the spread of the Omicron variant and the lack of data regarding this strain does not permit us to explore in depth his survival pattern. Secondly, the data reported by Kratzel et al. [38] seem to indicate a pattern of increasing survival on surfaces moving from winter to summer conditions, which overturns the evidence of the seasonal behaviour of the virus as noticed by Bueckert et al. [28]: “Anomalously, Kratzel et al. reported that SARS-CoV-2 was more stable on stainless steel at 30 °C than at 4 °C”. Furthermore, the results of Matson et al. [31], when placed in the general context, indicate a low survival sensitivity on surfaces to changing environmental conditions. Although a state of low specific enthalpy was examined, the half-lives associated with this setup remain very low, as if the peak survival was not appreciable. The same authors, comparing the results with a previous study [39], state: “The t_1/2_ we report here for SARS-CoV-2 in surface nasal mucus and sputum at 21 °C/40% (Table) is considerably shorter than what we found in culture media under similar conditions”. They do not mention the low-temperature state because the previous work [39] had analysed only one environmental condition, close to intermediate. Lastly, a different observation can be made about data published by Biryukov et al. [41]. Although not showing a marked peak, this profile grows up toward low enthalpies. However, a significant peak would still be compatible with the experimental data in this case. Its absence appears to be related simply to the lack of investigation on very low enthalpies. This clearly shows the practical utility of using the specific enthalpy as a physical quantity to steer experimental investigations.

The fit of the polynomial models shown in Figure 5 is worth being examined. For the *blood*, we have an R^2^ of approximately 0.96, but the F-test has a *p*-value greater than 0.05. The fitting to all *culture medium* test data (dashed grey line) is statistically significant but has an R^2^ less than 0.40. In contrast, when it is possible to regress the polynomial to the single author data, we found a significant F-test and R^2^ greater than 0.84. The regression to nasal mucus data presents a non-significant F-test and an R^2^ of approximately 0.28. The fitting to *saliva* and *simulated saliva* data shows an F-test with a *p*-value of 0.051 but an R^2^ of about 0.37. Regressions for *semen* and *sputum* show F-tests with a *p*-value of less than 0.05 or slightly higher, respectively, and an R^2^ of approximately 1. The fitting to tears test data give similar results to that for *blood*. The most significant analysis appears to be the one regarding the *culture medium* data, which confirms that there is a lot of noise when considering data from different sources. In contrast, the problem disappears as soon as only the tests performed by the same author are considered. A deeper insight into the SARS-CoV-2 data in *culture medium* may be then meaningful.

**Figure 6:**
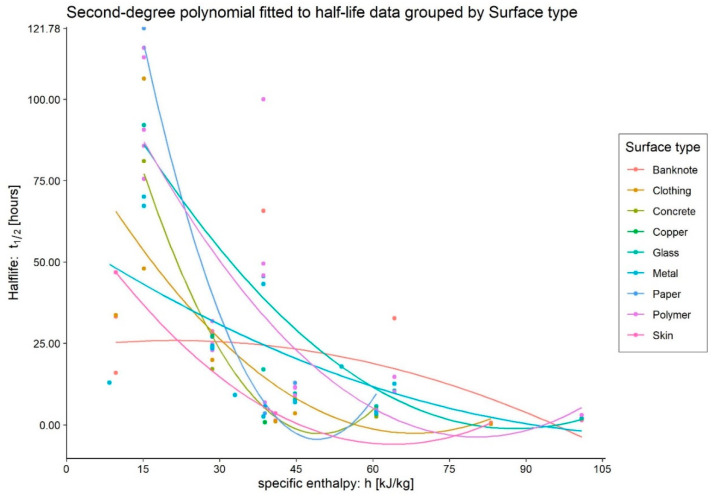
Comparison of SARS-CoV-2 survival data on surfaces in *Culture Medium* grouped by Surface Type.

In Figure 6, we can see that the data from experiments on banknotes show an atypical pattern. The polynomial regression model is non-significant on the F-test and has an R^2^ of approximately 0.21. Focussing on these data reveals that the measurements of Harbourt et al. [34], which report low half-lives at low temperatures when placed in the general context, force the fitting by inverting the concavity of the parabola. Here again, we can detect anomalous data compared to the typical behaviour of SARS-CoV-2 when varying environmental parameters.

**Figure 7:**
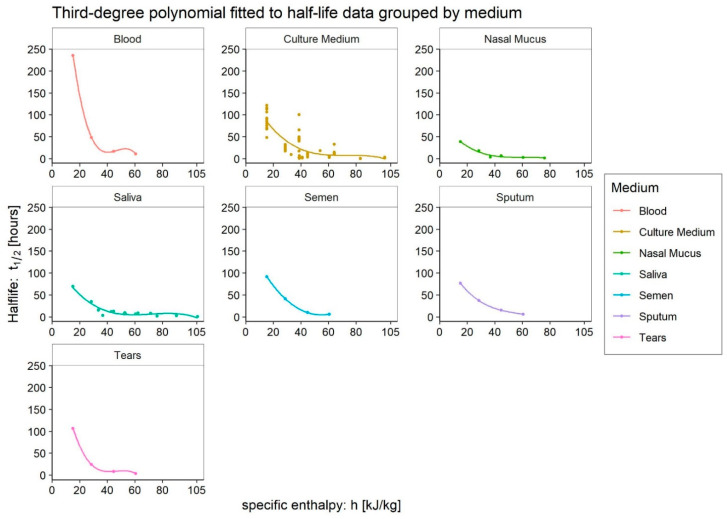
Comparison of survival behaviour in different media. *Blood* data have a very high pick at low specific enthalpy; this flattens the other plots, but the behavioural model appears to be still confirmed.

After removing anomalous data points that did not capture the behaviour of the virus at low specific enthalpies, and those produced by Pastorino et al. [29] with a high-protein medium in order to highlight the boost effect they have on virus survival, we can analyse all available data grouped by medium. We can fit a third-order polynomial, which can capture local minima and maxima, as shown in Figure 7. The third-order regressions for *blood*, *semen*, *sputum*, and *tears* cannot be statistically evaluated due to the limited number of points available. However, it is possible to appreciate the visualisation to understand whether the data agree with the general model. On the other hand, the regression to *culture medium* data is now statistically significant in general (F-test *p*-value << 0.001) with an R^2^ of approximately 0.57. The *nasal mucus* regression can still be considered significant given the *p*-value of 0.053 and an R^2^ of approximately 0.96. The regression to *saliva* data is also statistically significant (F-test *p*-value ≪ 0.001) with an R^2^ of approximately 0.92. These analyses confirm the results of the culture medium tests as those with the highest variability. This variability can be explained by the interference of the various substances of which the culture media are composed, but also by the higher number of available data sources. The borderline significance of the nasal mucus model may be explained by the smaller set of available data. The results of the regression analyses performed are shown in Table 2.

## 4. Discussion

At first, when analysing the survival data of viruses on surfaces, it must be emphasised that the laboratory procedures for obtaining the measurements suffer from high variability and poor standardisation. The problem is even more evident when compared with those used to measure aerosol survival.

**Figure 8:**
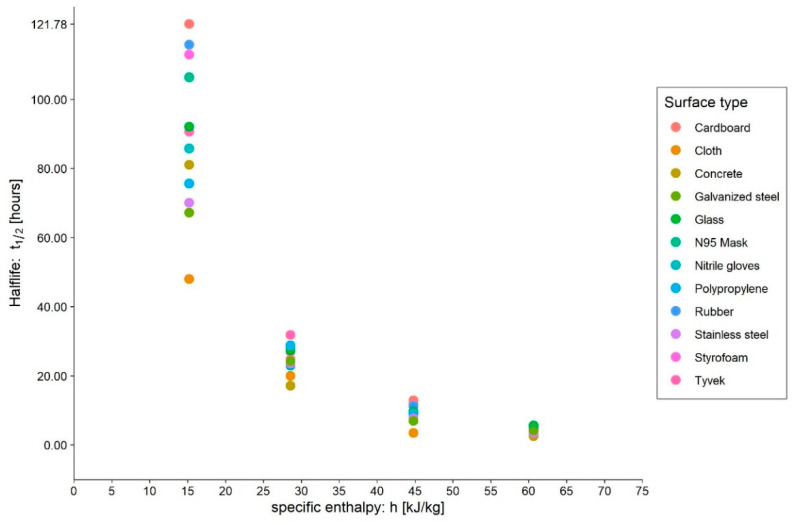
Scatterplot of SARS-CoV-2 survival behaviour on different surfaces. The elaboration was made based on data published by Kwon et al. [42].

**Figure 9:**
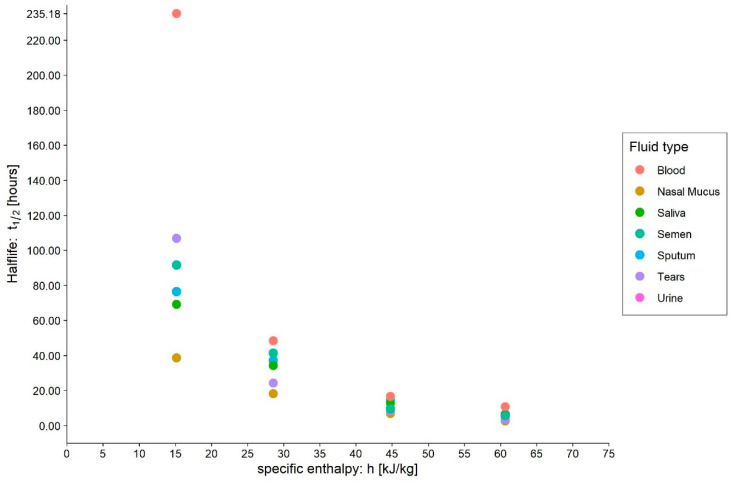
Scatterplot of SARS-CoV-2 survival behaviours in different biological fluids. Elaboration starting from the data published by Kwon et al. [27].

Taking note of the above evidence on the influence of the media used to carry out surface virus survival tests, the seasonal behaviour of SARS-CoV-2 also appears rather pronounced (Figure 5). This happens not only from a qualitative point of view but also from a quantitative one. As we can observe from the reprocessing of the data published by Kwon et al. [27,42] in Figure 8 and Figure 9, the dispersion of the measuring points does indeed increase as the specific enthalpy decreases. The dependence of the survival on the surface and fluid in which inoculation takes place is most pronounced at low enthalpies, that is, the region of best virus survival (Figure 8). In that range of specific enthalpies, the effects of different surfaces and media on SARS-CoV-2 survival should be investigated the most.

However, anomalous points are not lacking. The results of Matson et al. [31], although they confirm a general tendency toward a longer half-life under winter conditions when compared to other works, do not capture the intensity of increased virus survival for reasons probably linked to the laboratory setup. In the context of all the measurements analysed, these values are essentially anomalous with respect to the expected behaviour. This could be explained by the action of light, which can significantly reduce the survival of the virus in the local environment. However, since no laboratory control of light was mentioned in this work, this cannot be ruled out as a cause for more limited virus survival. Regarding the data published by Harbourt et al. [34], it should be noted that this is the only work that subjected the samples to storage at −80 °C before quantifying them. This different procedure and the possible action of light again, the control of which in their laboratory environment is here not specified, may explain the anomaly found. Lastly, the data published by Kratzel et al. [38]—in which the Bayesian calculation method adopted constitutes a further element of heterogeneity with respect to the other works—when analysed in the general context, are in contrast with all the different experiments and do not reveal the expected survival peak at low enthalpies. Once again, no explicit reference was made to the control of illumination during the experiments, so the effect of light could help to explain the absence of the peak.

In the following, a discussion of the results grouped by the medium is made.

### 4.1. Culture Medium

As expected, we can see in Figure 7 that the highest data dispersion affects the *culture medium* results. This dispersion appears to be due only in part to the greater number of data and, thus, to different laboratory measurement modalities, but mainly to the different compositions of the media used. Indeed, the composition of the culture media is not indifferent, as demonstrated by Pastorino et al. [29], and it constitutes an extensive source of noise.

The same figure shows how the Enthalpy method allows us to highlight the exact behaviour of the virus due to variations in environmental conditions. As a matter of findings, at low specific enthalpies, the absolute maximum of survival always occurs. At enthalpies of around 50–55 kJ/kg_dry-air_, we find a very pronounced local minimum; then the survival ability of SARS-CoV-2 tends to a slight local maximum or plateau, eventually reaching its absolute minimum for high enthalpies. However, it should be noted that negative enthalpies (values according to the calculation convention less than zero), which are representative outside of winters in very cold climates, and indoors of typical cold chain situations [46,47], have not been investigated. There are no specific studies on the effects—irreversible or temporary—of such extreme conditions on the virus, although they probably even occurred during storage procedures.

### 4.2. Blood

In the case of *blood* used as a medium (see Figure 7), it can be seen that the aforementioned local minimum is shifted slightly to the left (namely toward lower enthalpies). The possibility that this could be a peculiar behaviour of blood cannot be ruled out, but the data available are very few and provided by only one author. Indeed, the small dataset may have influenced our result. Four is, in fact, the minimum number of points required to regress a third-order polynomial. Such a limited number does not allow the unavoidable errors to be compensated for by repeated measurements and could explain the slight deviation of the minimum from the general model. In any case, it should be noted that the regression trend remains consistent with the general model. The high value of SARS-CoV-2 survival in this medium is worth noting.

### 4.3. Tears

The data collected using *tears* as a medium show (Figure 7) a local minimum shifted to the left. In this case, the saline pH of the tears could have interfered with the viability of the virus. The effect of pH on the survival of viruses, as well as the set of antimicrobial molecules present in these secretions, is actually known from the literature [48]. Again, the small number of points available to carry out the regression could explain this slight deviation. However, the general trend of the model is highlighted again, as in the case of *blood*.

### 4.4. Sputum

The same considerations regarding the number of data can be made when analysing survival in *sputum* (Figure 7). Once again, the deviation is slight and not in contrast with the general pattern. These results agree with those previously obtained by Spena et al. [1,2] and seem to trace a consistent pattern of SARS-CoV-2 behaviour, both in aerosols and on surfaces.

### 4.5. Saliva

The *saliva* facet in the plot (Figure 7) gathers data collected both in saliva and in simulated saliva. The regression performed showed a solid significance and a very high R^2^. The pattern observed is in accordance with the model hypothesised, where it is possible to note the peak at low enthalpy, the minimum in its aforementioned range and the tendency toward virus death with higher values. It is worth being noted that the maximum value of survival is similar to the others observed in the different media examined, with the exception already stated for blood.

### 4.6. Nasal Mucus

The same considerations made with regard to saliva are valid for the results of the model applied to the survival data in *nasal mucus*. The survival pattern is confirmed, and the statistical parameters reflect the minor availability of data points with a significance level borderline but a very high coefficient of determination. It should be noted that the value of the peak (namely toward lower enthalpies) is less prominent than for other media, suggesting an interfering role of the mucus [49,50].

### 4.7. Semen

The survival pattern of the virus in *semen* is in good accordance with the model (Figure 7) and it is possible to observe the peak at low enthalpy and the minimum in the confirmed range between 50 to 60 kJ/Kg_dry-air_. Again, the data availability is critical, so the coefficient of determination should be interpreted with caution. Nonetheless, the statical model showed a good level of significance.

### 4.8. Very Low Enthalpies

The dependence of survival on low-enthalpy environmental conditions (cold winters or cold chains) remains to be investigated [46,47]. Data analysis on the evolution of the pandemic confirmed how particularly severe climatic conditions generally hindered the spread of the virus. However, when moving from the analysis of contagions to the analysis of virus survival in a laboratory, consideration must be given to the possibility that very low temperatures could implement a kind of “conservation” for the virus. When the sample is moved from the climatic chamber to the station where the virus titration is performed, a recovery of virus activity may occur due to a temperature rise. Consideration must be given to the possibility that the absence of virus spread in very cold winters may be related more to a passive virus stasis or other confounding factors than to an actual reduction in virus survival.

### 4.9. Criticism of Measurements

The major limitation of this study consists, as already mentioned, rather than in the small availability of data, in the nonuniform laboratory procedures in which many confounding variables were involved.

Indeed, the freezing and de-freezing of microbiological samples covers many applications in the field of life and medical sciences. Numerous studies and protocols are dedicated to these procedures, aiming to: (i) make the process increasingly efficient; (ii) preserve the sample as best as possible, and (iii) minimise the loss of microorganism viability. Furthermore, numerous in vitro infection studies are conducted under optimal and controlled conditions for host cell growth (for human pathogenic microorganisms, around 37 °C). In contrast, a literature analysis shows that it is more complex to find homogeneous and comparable data, particularly with regard to the effect of low temperatures on the viability and infectivity of pathogenic microorganisms. The lack of standards mentioned above also precludes the development of calculation models that are more reliable and consistent with the virus survival assessment.

To enrich the availability of reliable experimental data to study the effect of environmental conditions on virus infection ability, it appears then necessary in the future to adopt suitable protocols aimed at ensuring the control of the temperature, relative humidity, and pressure parameters of human pathogenic microorganisms transmitted directly or indirectly, not only through bioaerosols but also on surfaces. In particular, it would be desirable to attain and share a universal standardised procedure that: (i) regulates the composition of the culture medium; (ii) solves the problem of unintended light interference; (iii) defines a single unit for the quantification of the virus on samples of different nature.

It is also essential to agree on the model more suited to describe the phenomena under consideration. Although models of biphasic decay are sometimes more appropriate, most works are based on the single-phase model. Therefore, to enhance opportunities for literature results comparison, it seems convenient to report half-lives for both models even when a biphasic model is considered more suited. This issue was also recently raised by Gracely [51] in a letter commenting on a paper by Hirose et al. [52], highlighting the difficulty the authors may have in describing the decay of the observed phenomenon when the monophasic model is not appropriate.

## 5. Conclusions

First of all, when dealing with the survival of coronaviruses on surfaces, it must be highlighted that the laboratory procedures for obtaining the measurements suffer from: (i) high variability, and (ii) poor standardisation. The problem is even more evident when compared with procedures used to measure survival in aerosols.

Indeed, the major limitation of the present study is in the source of the data: not having the opportunity to collect data through experiments with SARS-CoV-2 specifically aimed, we had to refer to experimental evidence found in previously peer-reviewed publications. This fact exposed our calculations to the aforementioned uncertainties, intrinsically occurring when comparing data from different sources but also, fortunately, allowed us to disclose this criticality.

Despite the difficulties above mentioned, the work carried out demonstrates that the detection of minimum viability of the SARS-CoV-2 virus on surfaces, and thus its probability of infecting susceptible exposed hosts, is possible and it occurs in an enthalpy range between 50 and 60 kJ/Kg_dry-air_. This range appears well-superimposed with the results we previously obtained from analyses in aerosols.

The present results also confirm that, while it remains impossible to clearly correlate the behaviour of SARS-CoV-2 with the variation in temperature or relative humidity independently, this becomes possible if the thermodynamic potential specific enthalpy is taken into account as an explanatory variable summarising the state of the environment in which virus survival is investigated.

Additionally, it sorts out evidence that the role of different surfaces becomes discriminating only in case of low enthalpies, i.e., in the range between 10 and 40 kJ/Kg_dry-air_, when the survival of SARS-CoV-2 generally increases; this means that mainly under these conditions the claimed difficulties with experimental procedures have to be addressed. More specifically, this refers to: (i) the medium used; (ii) the possible exposure of the samples to light; (iii) the pH of the experimental environment; (iv) the pressure at which the measurements are taken. These factors must then be kept under strict control, and their values must be reported in close association with the results; if not, the possibility of processing data from different sources will be lost.

Moreover, in order to improve comparisons from results in the literature, is relevant the general need for survival data standardisation. First of all, quantitatively, through a conventional unit for virus quantification in samples. However, also qualitatively: most works are based on the single-phase decay model, even though models of biphasic decay are sometimes more appropriate. That is, the half-lives of both models should be reported.

Lastly, as a further criticality in the case of the surface study of virus survival, the risk that a single investigation explores a small number of experimental points over a narrow range of specific enthalpy must be avoided. Measurements should investigate the survival of the virus by simultaneously diverging both temperature and relative humidity in a range of respective values sufficiently wide and consistent with the expected pattern of behaviour.

All of the above appears to be highly suitable and usefully decisive. Especially because, based on the evidence obtained, the Enthalpy approach is confirmed to be a simple, powerful, and robust method, as it can generally be extended to a broad context of different mechanisms of viral infection propagation.

## Figures and Tables

**Table 1:** Environmental characteristics of the laboratory setups.

	Strain	Medium	T	RH	h
			[°C]	[%]	[kJ/kg_dry-air_]
Harbourt et al. [34]	USA-WA1/2020	CM EMEM w/10% FBS	4	45	9.69
			22	45	40.95
			37	45	83.16
Kratzel et al. [38]	Munchen-1.1/2020/929	CM w/0.3% BSA	4	35	8.43
			20	35	33.00
			30	35	53.89
Pastorino et al. [29]	BavPat1/2020	CM w/5% FBS ^1^	20	50	38.58
Van Doremalen et al. [39]	nCoV-WA1-2020 Tor2	CM DMEM w/10% FBS	22	40	38.84
Matson et al. [31]	USA-WA1/2020	Nasal Mucus–Sputum	4	40	9.06
			21	40	36.82
			27	85	76.19
Biryukov et al. [40]	USA-WA1/2020	Simulated Saliva	24	20	33.54
			24	40	43.06
			24	60	52.68
			24	80	62.42
			28	40	52.28
			35	20	53.17
			35	40	71.53
			35	60	90.32
Biryukov et al. [41]	USA-WA1/2020	Simulated Saliva	54.5	20	105.60
Kwon et al. [27,42]	USA-WA1/2020	CM DMEM w/5% FBS Biological fluids	21	60	44.78
			25	70	60.67
			13	66	28.58
			5	75	15.20
Riddell et al. [43]	Betacoronavirus/Australia/SA01/2020	CM DMEM w/Penicillin, Streptomycin, Fungizone, 10% FCS and 1% BSA	20	50	38.58
			30	50	64.27
			40	50	100.91

T: temperature in Celsius degree; RH: relative humidity; h: specific enthalpy. CM: culture medium; EMEM: Eagle’s minimum essential medium; DMEM: Dulbecco’s modified eagle’s medium; FBS: foetal bovine serum; BSA: bovine serum albumin; FCS: foetal calf serum. ^1^ We used only the data from the experiment without BSA.

**Table 2:** Polynomial regression model compared before and after data review.

Medium	2nd-Degree Polynomial		3rd-Degree Polynomial	
	before		after	
	*p-Value*	*R* ^2^	*p-Value*	*R* ^2^
Blood	* 0.205	0.96	nd	nd
Culture medium	2.7 × 10^−10^	0.40	8.0 × 10^−15^	0.57
Nasal mucus	* 0.513	0.28	** 0.053	0.96
Saliva	0.051	0.37	2.7 × 10^−6^	0.92
Semen	0.011	1	nd	nd
Sputum	** 0.057	1	nd	nd
Tears	* 0.196	0.96	nd	nd

*p*-Value: F-statistic *p*-value; R^2^: coefficient of determination; nd: not done (not enough data to perform a regression). *: value greater than the level of significance (0.05); ** value slightly higher than the significance level established but acceptable in the context of the analysis.

## Data Availability

No new data were created or analysed in this study. Data sharing is not applicable to this article.

## References

[1] Spena A., Palombi L., Corcione M., Carestia M., Spena V.A. (2020). On the Optimal Indoor Air Conditions for SARS-CoV-2 Inactivation. An Enthalpy-Based Approach. Int. J. Environ. Res. Public Health.

[2] Spena A., Palombi L., Corcione M., Quintino A., Carestia M., Spena V.A. (2020). Predicting SARS-CoV-2 Weather-Induced Seasonal Virulence from Atmospheric Air Enthalpy. Int. J. Environ. Res. Public Health.

[3] Casanova L.M., Jeon S., Rutala W.A., Weber D.J., Sobsey M.D. (2010). Effects of Air Temperature and Relative Humidity on Coronavirus Survival on Surfaces. Appl. Environ. Microbiol..

[4] Azuma K., Yanagi U., Kagi N., Kim H., Ogata M., Hayashi M. (2020). Environmental factors involved in SARS-CoV-2 transmission: Effect and role of indoor environmental quality in the strategy for COVID-19 infection control. Environ. Health Prev. Med..

[5] Chin A.W.H., Chu J.T., Perera M.R., Hui K.P., Yen H.L., Chan M.C., Peiris M., Poon L.L. (2020). Stability of SARS-CoV-2 in different environmental conditions. Lancet Microbe.

[6] Liu Y., Li T., Deng Y., Liu S., Zhang D., Li H., Wang X., Jia L., Han J., Bei Z. (2021). Stability of SARS-CoV-2 on environmental surfaces and in human excreta. J. Hosp. Infect..

[7] Grinchuk P.S., Fisenko K.I., Fisenko S.P., Danilova-Tretiak S.M. (2021). Isothermal Evaporation Rate of Deposited Liquid Aerosols and the SARS-CoV-2 Coronavirus Survival. Aerosol Air Qual. Res..

[8] Kasloff S.B., Leung A., Strong J.E., Funk D., Cutts T. (2021). Stability of SARS-CoV-2 on critical personal protective equipment. Sci. Rep..

[9] Behzadinasab S., Chin A., Hosseini M., Poon L., Ducker W.A. (2020). A Surface Coating that Rapidly Inactivates SARS-CoV-2. ACS Appl. Mater. Interfaces.

[10] REALM Project-Test 1 Results Available|OCLC. https://www.oclc.org/realm/news/20200622-round-1-test-results-now-available.html.

[11] REALM Project-Test 2 Results. https://www.oclc.org/realm/news/20200720-test2-results-available.html.

[12] REALM Project-Test 3 Results. https://www.oclc.org/realm/news/20200818-test-3-results-available.html.

[13] Akers T.G., Hers J.F.P., Winkler K.C. (1973). Some aspects of the airborne inactivation of viruses. Airborne Transmission and Airborne Infection.

[14] de Jong J.C. (1970). Decay mechanism of polio and EMC viruses in aerosols. Third International Symposium on Aerobiology.

[15] Morawska L. (2006). Droplet fate in indoor environments, or can we prevent the spread of infection?. Indoor Air.

[16] Gershenson A., Gierasch L.M. (2011). Protein folding in the cell: Challenges and progress. Curr. Opin. Struct. Biol..

[17] Ijaz M.K., Brunner A.H., Sattar S.A., Nair R.C., Johnson-Lussenburg C.M. (1985). Survival Characteristics of Airborne Human Coronavirus 229E. J. Gen. Virol..

[18] Ikäheimo T.M., Jaakkola K., Jokelainen J., Saukkoriipi A., Roivainen M., Juvonen R., Vainio O., Jaakkola J.J. (2016). A Decrease in Temperature and Humidity Precedes Human Rhinovirus Infections in a Cold Climate. Viruses.

[19] Davis R.E., Dougherty E., McArthur C., Huang Q.S., Baker M.G. (2016). Cold, dry air is associated with influenza and pneumonia mortality in Auckland, New Zealand. Influenza Other Respir. Viruses.

[20] Cai J., Sun W., Huang J., Gamber M., Wu J., He G. (2020). Indirect Virus Transmission in Cluster of COVID-19 Cases, Wenzhou, China, 2020. Emerg. Infect. Dis..

[21] Yang W., Marr L.C. (2012). Mechanisms by which ambient humidity may affect viruses in aerosols. Appl. Environ. Microbiol..

[22] Yang W., Elankumaran S., Marr L.C. (2012). Relationship between Humidity and Influenza A Viability in Droplets and Implications for Influenza’s Seasonality. PLoS ONE.

[23] Ratnesar-Shumate S., Williams G., Green B., Krause M., Holland B., Wood S., Bohannon J., Boydston J., Freeburger D., Hooper I. (2020). Simulated Sunlight Rapidly Inactivates SARS-CoV-2 on Surfaces. J. Infect. Dis..

[24] Raiteux J., Eschlimann M., Marangon A., Rogée S., Dadvisard M., Taysse L., Larigauderie G. (2021). Inactivation of SARS-CoV-2 by Simulated Sunlight on Contaminated Surfaces. Microbiol. Spectr..

[25] Herman J., Biegel B., Huang L. (2021). Inactivation times from 290 to 315 nm UVB in sunlight for SARS coronaviruses CoV and CoV-2 using OMI satellite data for the sunlit Earth. Air Qual. Atmos. Health.

[26] American Society of Heating Refrigerating Air Conditioning Engineers (2022). ASHRAE Positions on Infectious Aerosols. www.ashrae.org.

[27] Kwon T., Gaudreault N.N., Richt J.A. (2021). Seasonal Stability of SARS-CoV-2 in Biological Fluids. Pathogens.

[28] Bueckert M., Gupta R., Gupta A., Garg M., Mazumder A. (2020). Infectivity of SARS-CoV-2 and Other Coronaviruses on Dry Surfaces: Potential for Indirect Transmission. Materials.

[29] Pastorino B., Touret F., Gilles M., De Lamballerie X., Charrel R.N. (2020). Prolonged Infectivity of SARS-CoV-2 in Fomites. Emerg. Infect. Dis..

[30] Szpiro L., Pizzorno A., Durimel L., Julien T., Traversier A., Bouchami D., Marie Y., Rosa-Calatrava M., Terrier O., Moules V. (2020). Role of interfering substances in the survival of coronaviruses on surfaces and their impact on the efficiency of hand and surface disinfection. medRxiv.

[31] Matson M.J., Yinda C.K., Seifert S.N., Bushmaker T., Fischer R.J., van Doremalen N., Lloyd-Smith J.O., Munster V.J. (2020). Effect of Environmental Conditions on SARS-CoV-2 Stability in Human Nasal Mucus and Sputum. Emerg. Infect. Dis..

[32] Mishra R., Krishnamoorthy P., Gangamma S., Raut A.A., Kumar H. (2020). Particulate matter (PM10) enhances RNA virus infection through modulation of innate immune responses. Environ. Pollut..

[33] Groulx N., Urch B., Duchaine C., Mubareka S., Scott J.A. (2018). The Pollution Particulate Concentrator (PoPCon): A platform to investigate the effects of particulate air pollutants on viral infectivity. Sci. Total Environ..

[34] Harbourt D.E., Haddow A.D., Piper A.E., Bloomfield H., Kearney B.J., Fetterer D., Gibson K., Minogue T. (2020). Modeling the stability of severe acute respiratory syndrome coronavirus 2 (SARS-CoV-2) on skin, currency, and clothing. PLOS Negl. Trop. Dis..

[35] American Society of Heating Refrigerating Air Conditioning Engineers (2005). ASHRAE Handbook of Fundamentals.

[36] Hyland R.W., Wexter A. (1983). Formulations for the thermodynamic properties of the saturated phases of H_2_O from 173.15 K to 473.15 K. ASHRAE Trans..

[37] Wulff N.H., Tzatzaris M., Young P.J. (2012). Monte Carlo simulation of the Spearman-Kaerber TCID50. J. Clin. Bioinform..

[38] Kratzel A., Steiner S., Todt D., V’Kovski P., Brueggemann Y., Steinmann J., Steinmann E., Thiel V., Pfaender S. (2020). Temperature-dependent surface stability of SARS-CoV-2. J. Infect..

[39] Van Doremalen N., Bushmaker T., Morris D.H., Holbrook M.G., Gamble A., Williamson B.N., Tamin A., Harcourt J.L., Thornburg N.J., Gerber S.I. (2020). Aerosol and Surface Stability of SARS-CoV-2 as Compared with SARS-CoV-1. N. Engl. J. Med..

[40] Biryukov J., Boydston J.A., Dunning R.A., Yeager J.J., Wood S., Reese A.L., Ferris A., Miller D., Weaver W., Zeitouni N.E. (2020). Increasing Temperature and Relative Humidity Accelerates Inactivation of SARS-CoV-2 on Surfaces. mSphere.

[41] Biryukov J., Boydston J.A., Dunning R.A., Yeager J.J., Wood S., Ferris A., Miller D., Weaver W., Zeitouni N.E., Freeburger D. (2021). SARS-CoV-2 is rapidly inactivated at high temperature. Environ. Chem. Lett..

[42] Kwon T., Gaudreault N.N., Richt J.A. (2021). Environmental Stability of SARS-CoV-2 on Different Types of Surfaces under Indoor and Seasonal Climate Conditions. Pathogens.

[43] Riddell S., Goldie S., Hill A., Eagles D., Drew T.W. (2020). The effect of temperature on persistence of SARS-CoV-2 on common surfaces. Virol. J..

[44] Guo L., Yang Z., Zhang L., Wang S., Bai T., Xiang Y., Long E. (2021). Systematic review of the effects of environmental factors on virus inactivation: Implications for coronavirus disease 2019. Int. J. Environ. Sci. Technol..

[45] Cappi R., Casini L., Tosi D., Roccetti M. (2022). Questioning the seasonality of SARS-COV-2: A Fourier spectral analysis. BMJ Open.

[46] Lu L., Quintela I., Lin C., Lin T., Lin C., Wu V.C.H., Lin C. (2021). A review of epidemic investigation on cold-chain food-mediated SARS-CoV-2 transmission and food safety consideration during COVID-19 pandemic. J. Food Saf..

[47] Chi Y., Zheng S., Liu C., Wang Q. (2021). Transmission of SARS-CoV-2 on cold-chain food overpacks: A new challenge. J. Glob. Health.

[48] McDermott A.M. (2013). Antimicrobial compounds in tears. Exp. Eye Res..

[49] Wardzala C.L., Wood A.M., Belnap D.M., Kramer J.R. (2022). Mucins Inhibit Coronavirus Infection in a Glycan-Dependent Manner. ACS Central Sci..

[50] Linden S.K., Sutton P., Karlsson N.G., Korolik V., McGuckin M.A. (2008). Mucins in the mucosal barrier to infection. Mucosal Immunol..

[51] Gracely E.J. (2021). Half-Life Defined as the Time Until Half of the Log Titer Is Not Informative and Should Be Replaced With Segments: Comment on Hirose et al., 2020. Clin. Infect. Dis..

[52] Hirose R., Ikegaya H., Naito Y., Watanabe N., Yoshida T., Bandou R., Daidoji T., Itoh Y., Nakaya T. (2021). Survival of Severe Acute Respiratory Syndrome Coronavirus 2 (SARS-CoV-2) and Influenza Virus on Human Skin: Importance of Hand Hygiene in Coronavirus Disease 2019 (COVID-19). Clin. Infect. Dis..

